# Transcranial direct current stimulation for depression in Alzheimer’s disease: study protocol for a randomized controlled trial

**DOI:** 10.1186/s13063-017-2019-z

**Published:** 2017-06-19

**Authors:** Zui Narita, Yuma Yokoi

**Affiliations:** Department of Psychiatry, National Center Hospital, National Center of Neurology and Psychiatry, 4-1-1, Ogawahigashi, Kodaira, Tokyo, Japan

**Keywords:** Transcranial direct current stimulation, Brain stimulation, Depression, Alzheimer’s disease

## Abstract

**Background:**

Patients with Alzheimer’s disease frequently elicit neuropsychiatric symptoms as well as cognitive deficits. Above all, depression is one of the most common neuropsychiatric symptoms in Alzheimer’s disease but antidepressant drugs have not shown significant beneficial effects on it. Moreover, electroconvulsive therapy has not ensured its safety for potential severe adverse events although it does show beneficial clinical effect. Transcranial direct current stimulation can be the safe alternative of neuromodulation, which applies weak direct electrical current to the brain. Although transcranial direct current stimulation has plausible evidence for its effect on depression in young adult patients, no study has explored it in older subjects with depression in Alzheimer’s disease. Therefore, we present a study protocol designed to evaluate the safety and clinical effect of transcranial direct current stimulation on depression in Alzheimer’s disease in subjects aged over 65 years.

**Method:**

This is a two-arm, parallel-design, randomized controlled trial, in which patients and assessors will be blinded. Subjects will be randomized to either an active or a sham transcranial direct current stimulation group. Participants in both groups will be evaluated at baseline, immediately, and 2 weeks after the intervention.

**Discussion:**

This study investigates the safety and effect of transcranial direct current stimulation that may bring a significant impact on both depression and cognition in patients with Alzheimer’s disease, and may be useful to enhance their quality of life.

**Trial registration:**

ClinicalTrials.gov, NCT02351388. Registered on 27 January 2015. Last updated on 30 May 2016.

**Electronic supplementary material:**

The online version of this article (doi:10.1186/s13063-017-2019-z) contains supplementary material, which is available to authorized users.

## Background

Dementia is a disorder that is characterized by a decline in cognition in one or more cognitive domains such as learning and memory, language, executive function, complex attention, perceptual-motor, and social cognition [1]. According to the World Alzheimer Report 2015, 36 million people were living with dementia in 2010, nearly doubling every 20 years to 66 million by 2030 and to 115 million by 2050 [2]. Alzheimer’s disease (AD), the most common form of dementia in older people, is increasingly prevalent with advancing age, and the overall burden of it is substantial worldwide [3–8]. Cognitive dysfunction in AD reduces the quality of life (QOL) of patients and caregivers [9]. At the same time, neuropsychiatric symptoms (NPS) in AD compromise their QOL [10]. Depressive mood is one of the most common NPS in AD as well as agitation and apathy [11]. The prevalence rate of depression in AD is from 36.7 [12] to 47.8% [11].

However, antidepressant drugs have not shown significant beneficial effect on depression in AD. A meta-analysis demonstrated that selective serotonin reuptake inhibitors and serotonin and norepinephrine reuptake inhibitors did not show significant beneficial effect on depression in AD [13]. In addition, a randomized controlled trial (RCT) with large sample, revealed that neither sertraline nor mirtazapine showed superiority to placebo, and concluded that antidepressants should not be used first for depression in AD [14].

Electroconvulsive therapy (ECT) is one of the most promising nonpharmacological treatments for depression [15, 16]. A review article suggested that ECT does show beneficial effect in both older patients over 65 years, and in patients with dementia, but it did not conclude that ECT is a safe treatment for them [17]. A case series showed that ECT did not induce long-term cognitive deficits in older patients, but the result was not easily generalized because of confounders [18]. A chart review demonstrated that ECT produced delirium in 49% of patients with AD [19]. Moreover, a secondary analysis of data collected from a large prospective cohort study suggested that delirium can accelerate the trajectory of cognitive decline in patients with AD [20].

Transcranial direct current stimulation (tDCS) is a simple, cheap, and safe method of neuromodulation, based on the application of weak, direct electrical current to the brain through relatively large electrodes. Two electrodes are placed over the scalp, in which anodal and cathodal stimulation increases and decreases cortical excitability, respectively [21]. One RCT, in which the anodal electrode was placed over the left dorsolateral prefrontal cortex (DLPFC), showed significant effect of tDCS on the Montgomery-Asberg Depression Rating Scale (MADRS) in major depressive disorder [22]. Furthermore, another RCT, using a factorial design to sertraline/placebo and active/sham tDCS to the left DLPFC, verified that there was a significant difference in MADRS scores when comparing the combined treatment group (sertraline/active tDCS) versus sertraline only, and tDCS only versus placebo/sham tDCS [23]. In addition, a meta-analysis of individual patient data concluded that the effect size of tDCS is comparable with those reported for repetitive transcranial magnetic stimulation and antidepressant drug treatment in primary care [24]. The antidepressant effects of tDCS are based on the finding that the left DLPFC is hypoactive in depression and, hence, anodal tDCS would possibly restore prefrontal activity by increasing activity in this area [25]. One RCT demonstrated that in the motor cortex of healthy volunteers, tDCS altered cortical excitability more effectively when given daily rather than on alternate days over a 5-day period [26].

However, no study has evaluated the safety and effect of tDCS on depression in AD although one RCT assessed the effect of tDCS on apathy in AD, which did not show significant effect, possibly because of a small number of interventions (six sessions of tDCS in 2 weeks) [27]. Therefore, we present a study protocol designed to evaluate the safety and effect of tDCS on depression in AD patients over 65 years.

## Method

### Study design

**Fig. 1:**
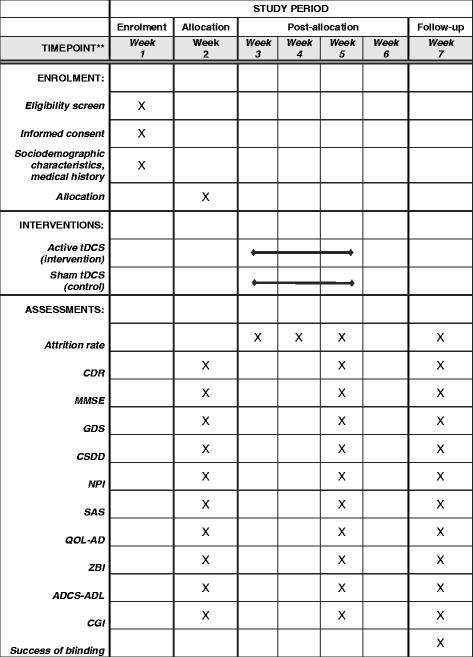
Schedule of enrollment, interventions, and assessments. *tDCS* transcranial direct current stimulation, *CDR* Clinical Dementia Rating, *MMSE* Mini Mental State Examination, *GDS* Geriatric Depression Scale, *CSDD* Cornell Scale for Depression in Dementia, *NPI* Neuropsychiatric Inventory, *SAS* Starkstein Apathy Scale, *QOL-AD* Quality of Life-Alzheimer’ disease, *ZBI* Zarit Burden Interview, *ADCS-ADL* Alzheimer’s Disease Cooperative Study Activities of Daily Living, *CGI* Clinical Global Impression

This protocol is presented in accordance with the 2013 SPIRIT (Standard Protocol Items: Recommendations for Interventional Trials) Statement (See Additional file 1 for the populated SPIRIT Checklist and Fig. 1 for the trial schedule of enrollment, interventions, and assessments in accordance with recommended SPIRIT figure) which was developed to provide guidance in the form of a checklist of recommended items to include in a clinical trial protocol to help improve its content and quality [28]. This is a single-center trial, which will be conducted at the National Center of Neurology and Psychiatry, Tokyo, Japan. This is a two-arm, parallel-design, double-blind RCT in which patients and assessors will be blinded. Subjects will be randomized with a 1:1 ratio to either the active or the sham tDCS group with a computer-generated sequence. The superiority of active tDCS to sham tDCS will be investigated. Allocation concealment will be maintained with sealed opaque envelopes. Subjects will receive 15 consecutive 30-min applications of active/sham tDCS from Monday to Friday for 3 weeks. A participant’s allocated intervention during the trial will be revealed by the principle investigator after the study endpoint. The trial results will be communicated by the study coordinators when requested.

### Participants

Patients enrolled in the study must follow all of the inclusion criteria below:

1. Participants who meet the criteria of probable AD defined by National Institute of Neurological and Communicative Diseases and Stroke-Alzheimer’s Disease and Related Disorders Association (NINCDS-ADRDA) research criteria [29]
2. Participants who meet the criteria of depression in AD defined by National Institute of Mental Health (NIMH) criteria [30]
3. Participants who have been on a fixed dose (including participants without any prescription) of antidepressants for at least 2 weeks on the screening visit
4. Participants who have been on a fixed dose (including participants not prescribed) of antidementia drugs (donepezil, rivastigmine, galantamine, and memantine) for at least 4 weeks on the screening visit
5. Ambulatory participants with or without any aiding device
6. Participants who have a study partner, who lives with them for at least 10 h a week to report their behavior
7. Participants who sign and give consent, or whose family member provides consent on their behalf
8. Participants who are community dwelling
9. Participants who are over 65 years old

Patients with any of the following criteria will be excluded from the study:

1. People whose cognitive deficits are better explained by another disease such as cerebral infarction, Parkinson disease, multiple sclerosis, and normotensive hydrocephalus
2. A history of epilepsy
3. Current treatment of antipsychotic medication
4. An urgent risk of suicide or severe depression and need to be hospitalized to psychiatric units
5. A history of ineffectiveness with ECT or tDCS
6. People who are clinically contraindicated to receive ECT or tDCS such as having a tattoo or metal embedded in their scalp or brain
7. Current treatment with benzodiazepines or antiepileptic drugs
8. People who scored less than 10 on the Mini Mental State Examination (MMSE) [31] or who scored 3 or more on the Clinical Dementia Rating Scale (CDR) [32]
9. People who scored less than 6 on the 15-item Geriatric Depression Scale (GDS) [33] at baseline
10. People who are unable to undergo a video recording on the evaluation interview
11. People who are diagnosed with concurrent delirium by clinicians.

### Interventions

Direct current will be transferred by 35-cm^2^ saline-soaked sponge electrodes and delivered by Soterix Medical 1 x 1 Transcranial Direct Current Low-Intensity Stimulator Model 1300A. For each session, the tDCS montage will comprise placement of the anode over the left DLPFC and the cathode over the contralateral supraorbital area which corresponds to the F3 and FP2 areas, according to the International 10–20 electroencephalography system. We will apply direct current of 2 mA for 30 min/day, for 15 days over three consecutive weeks. The dose and frequency of stimulation was chosen based on previous research for depression in young adults [22].

For the sham group, the device will be turned off after 1 min of active stimulation. The electrode position and the other procedures to set up, including electrode moisture, checking the contact, will be identical. The display on the device will be kept outside participants’ visual fields as the device will be turned off without subjects noticing. A controlled study demonstrated that blinding integrity of tDCS and pharmacological treatment were comparable [34]. The raters and patients will be blinded to the treatment, and the contact between participants will be avoided to enhance the effect of study blinding.

A trained psychiatrist will administer the tDCS intervention. Since the experimenter will not be blinded, their interaction with participants will be minimized. Also, the experimenter will not participate in the assessment of outcomes or in any other aspect of the trial.

Allocated interventions will be discontinued according to the following criteria:

1. In case patients cease giving their informed consent to participate
2. In case a severe adverse effect is observed
3. In case patients fail to undergo five consecutive sessions of tDCS

In order to improve adherence, we will provide all included patients and their study partners with costs of transportation, and will remind and reschedule all of the patients’ visits if necessary.

The following interventions will be restricted and recognized as protocol deviation during the trial:

1. Adjusting the dose of antidepressant drugs except trazodone or mianserin
2. Adjusting the dose of antidementia drugs
3. Antiepileptic drugs
4. Adjusting the dose of lithium
5. Quetiapine over a dose of 50 mg/day
6. Trazodone over a dose of 50 mg/day
7. Mianserin over a dose of 20 mg/day
8. Adjusting the dose of antipsychotic medication except quetiapine
9. Benzodiazepines

### Outcome measures

**Fig. 2:**
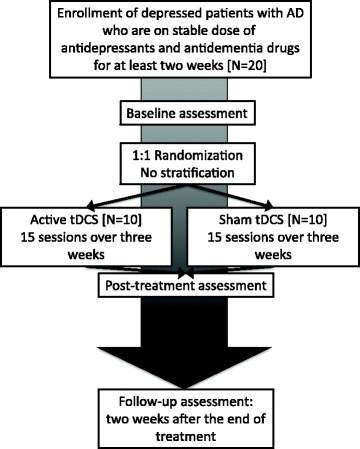
Flowchart summarizing the trial

Patients will be assessed after being informed of the objectives of the study and giving their informed consent to participate (see Additional files 2 and 3). Data will be collected following an assessment that will be implemented at baseline, immediately, and 2 weeks after the end of treatment (see Fig. 2). Baseline and follow-up evaluations will be performed by experienced psychiatrists blinded to group assignments.

The primary outcome measure is attrition rate caused by any adverse event since no study has evaluated the safety of tDCS on depression in AD. Secondary outcome measures include the MMSE, the CDR, the GDS, the Cornell Scale for Depression in Dementia (CSDD) [33], the Neuropsychiatric Inventory (NPI) [35], the Starkstein Apathy Scale (SAS) [36], the Quality of Life-Alzheimer’s Disease (QOL-AD) [37], the Zarit Burden Interview (ZBI) [38], the Alzheimer’s Disease Cooperative Study-Activities of Daily Living (ADCS-ADL) [39], and the Clinical Global Impression (CGI) [40]. The MMSE is an objective scale developed to provide a brief screening test that quantitatively assesses the severity of cognitive impairment and documents cognitive changes occurring over time [31]. The CDR is an objective scale used to characterize six domains of cognitive and functional performance applicable to Alzheimer’s disease and related dementias [32]. The GDS (self-report) and the CSDD (objective scale) are both valid screening tools for depression in older people [33]. The NPI is a proxy-reported scale developed to assess 12 neuropsychiatric disturbances common in dementia [35]. The SAS is a self-reported scale recommended to screen for, and measure, the severity of apathetic symptoms [36]. The QOL-AD is one of the most widely used self-reported dementia-specific QOL instruments in the world [37]. The ZBI is a valid and reliable caregiver-reported instrument for measuring the burden of them [38]. The ADCS-ADL is a proxy-rated scale developed to assess the ability of patients with moderate to severe dementia to perform activities of daily living [39]. The CGI is a tool used to offer a readily understood, practical measurement tool that can easily be administered by a clinician in a busy clinical practice setting [40]. To ensure the success of blinding, we will ask participants and outcome assessors at the endpoint to guess whether the treatment was active or sham.

The schedule of enrollment, interventions, and assessments is summarized in Fig. 1. Participants will be recruited by referrals of two experienced psychiatrists (ZN and YY) in the National Center of Neurology and Psychiatry. This is a pilot study to inform the design of a future full-scale randomized trial and aims to test the feasibility of conducting the study. Recruitment of a total of 20 participants, 10 for the active tDCS group and 10 for the sham tDCS group, respectively, will be deemed sufficient for a pilot study and will be affordable in our research setting. We will use a blocked randomization process, with random 4-size and 6-size blocks, in order to maintain an adequate balance in the number of subjects allocated to both groups. We expect that one or two patients can be recruited per month on average, and that it will be possible to recruit 20 participants in 20 months.

All assessors will be trained at a workshop every month. All data will be administered in the Electronic Data Capture (EDC) system. Allocation and other identifiable data of participants will be stored in a computer which does not have Internet access. Only the study coordinators and the Data Monitoring Committee can open the EDC system using passwords.

### Statistical analysis

Statistical analysis on outcome measures will be conducted using STATA. We will handle missing data with a multiple imputation method as an intention-to-treat (ITT) analysis for participants who received at least one session of tDCS. We will also perform a per-protocol approach as a sensitivity analysis for the comparison of the results. For the MMSE, the CDR, the GDS, the CSDD, the NPI, the SAS, the QOL-AD, the ZBI, and the ADCS-ADL, we will use analysis of covariance (ANCOVA) regarding age, sex, severity of depression, and severity of AD at baseline as covariates. For the CGI, we will use the Wilcoxon signed-rank test. With alpha of 0.05, a two-sided *p* value, and power of 0.8, a 7-point CSDD difference (standard deviation = 5) will be required to create significance between groups [14].

### Monitoring

A systematic review has demonstrated that the most common adverse events from tDCS are itching, tingling, headache, burning sensation, and discomfort [41]. A trained psychiatrist will check for adverse effects during/immediately after every session and evaluate the safety every week during the intervention. An independent Safety Monitoring Committee will run an interim analysis for safety when the tenth participant finishes the schedule, and will decide if the trial has to be terminated or modified. An independent Monitoring Committee will monitor the data in the EDC system every week.

## Discussion

Depression is one of the most frequent NPS in patients with AD, and has a negative impact on their QOL [10]. However, neither antidepressant drugs nor ECT have been established as a persuasive treatment for it because the former has not shown significant effect [13, 14] and the latter has not ensured its safety [17–20].

As stated before, tDCS is a safe method of brain stimulation and has a plausible evidence for treating depression in young patients [22–24]. Given this, tDCS may be also potentially useful to ameliorate depression in older patients with AD. Nevertheless, so far no RCT has been performed for tDCS in patients with depression in AD. The RCT described in this paper concerns the safety and effect of neuromodulation by weak direct current that may have a significant impact on both depression and cognition, and may be useful for patients with depression in AD to enhance their QOL.

The topic of adherence may be regarded as a potential pitfall in this protocol. However, as mentioned before, by adding costs of transportation for all included patients and their study partners to the study budget, we plan to compensate for this situation. Also, the study coordinator will remind and reschedule all of the patients’ visits as needed.

Another limitation in this trial would be the small sample. Since this is a pilot study to inform the design of a future full-scale randomized trial and aims to test the feasibility of conducting the study, we estimated that a sample size of 20 will be sufficient and will be affordable in terms of feasibility in recruitment and study implementation. We will perform a blocked randomization to equalize the number of subjects in each group but there may be still imbalance in baseline characteristics between groups which will compromise the internal validity. Furthermore, even if tDCS shows significant effect on several outcome measures in this trial, we may not immediately generalize the result to the wider population because the sample may not directly represent it which is an issue of external validity. Hence, further RCTs with larger samples and additional evaluations will be needed.

We believe that this trial is a well-designed RCT that will investigate tDCS intervention in a way which has not yet been evaluated. Even if the results do not prove our hypothesis, the gathered data will contribute to a field which has not been widely studied.

### Trial status

This study is currently recruiting participants.

## Additional files

Additional file 1: SPIRIT (Standard Protocol Items: Recommendations for Interventional Trials) 2013 Checklist: recommended items to address in a clinical trial protocol and related documents. (DOC 121 kb)

Additional file 2: Consent Form (translated to English). (DOC 44 kb)

Additional file 3: Information Sheet (translated to English). (DOCX 424 kb)

## Abbreviations

AD: Alzheimer’s disease
ADCS-ADL: Alzheimer’s Disease Cooperative Study Activities of Daily Living
ANCOVA: Analysis of covariance
CDR: Clinical Dementia Rating
CGI: Clinical Global Impression
CSDD: Cornell Scale for Depression in Dementia
DLPFC: Dorsolateral prefrontal cortex
ECT: Electroconvulsive therapy
EDC: Electronic Data Capture
GDS: Geriatric Depression Scale
ITT: Intention-to-treat
MADRS: Montgomery-Asberg Depression Rating Scale
MMSE: Mini Mental State Examination
NIMH: National Institute of Mental Health
NINCDS-ADRDA: National Institute of Neurological and Communicative Diseases and Stroke-Alzheimer’s Disease and Related Disorders Association
NPI: Neuropsychiatric Inventory
NPS: Neuropsychiatric symptoms
QOL: Quality of life
QOL-AD: Quality of Life-Alzheimer’ disease
RCT: Randomized controlled trial
SAS: Starkstein Apathy Scale
SPIRIT: Standard Protocol Items: Recommendations for Interventional Trials
ZBI: Zarit Burden Interview
