# Supplementary material for: Transcranial direct current stimulation for depression in Alzheimer’s disease: study protocol for a randomized controlled trial
Source: Trials. 2017 Jun 19;18:285. doi: 10.1186/s13063-017-2019-z (PMC5477338; doi:10.1186/s13063-017-2019-z)
Supplement: Supplementary file 2 — Consent Form (translated to English). (DOC 44 kb) [file 13063_2017_2019_MOESM2_ESM.doc]

**Concent form**

To Principle Investigator, Yuma Yokoi, MD.,

　I got a full explanation from _________ (explainer) about the study called " Ameliorating Depression in Alzheimer’s disease Patients by Transcranial direct current stimulation (ADAPT) – preliminary research ",using explanatory documents and understood the purpose and method of research, the next research cooperation matters that I cooperate with and the disadvantages. Therefore, I agree to participate in this research by my free will.

Items fully explained and informed

□1 Purpose of this study

□2 Right to voluntarily participate and withdraw consent

□2-1 After you have agreed to participate in this research, you can withdraw from participating in research freely without being disadvantaged at any time

□2-2 If you refuse to participate, for that reason you will not suffer any disadvantages for future treatment

□3 Research execution method and participation cooperation matter

　□3-1 Video shooting

□4 Risk and benefit when participating in the study

□5 Privacy protection

□6 Use of research results

□7 Costs

□8 Disclosure of research plan and personal information

□9 Dissemination of research results

□10 Funding source relating to this study

□11 Clinical trial insurance

□12Agreement by surrogates

Participants

Date

Signature

Address

Telephone

Surrogates

Date

Signature 　　　　　　　　　　　　　　　　　　（Relationship 　　　　　　）

Explainer

I gave an explanation based on the explanatory document of this research when obtaining subject consent for this research.

Date

Signature

National Center of Neurology and Psychiatry
