# Supplementary material for: Transcranial direct current stimulation for depression in Alzheimer’s disease: study protocol for a randomized controlled trial
Source: Trials. 2017 Jun 19;18:285. doi: 10.1186/s13063-017-2019-z (PMC5477338; doi:10.1186/s13063-017-2019-z)
Supplement: Supplementary file 3 — Information Sheet (translated to English). (DOCX 424 kb) [file 13063_2017_2019_MOESM3_ESM.docx]

Instructions to patients and family members for " Ameliorating Depression in Alzheimer’s disease Patients by Transcranial direct current stimulation (ADAPT) – preliminary research "

Principle Investigator: Yuma Yokoi, MD

Sponsor: National Center of Neurology and Psychiatry

Department of Psychiatry

ICF version 2.2 (Oct 14^th^, 2014)

This document explains about "transcranial direct current electrical stimulation test for depression of Alzheimer's disease" and it is a material for you to decide whether you participate or not.

**Introduction**

In Alzheimer's disease, it is known that not only a decline in cognitive function but also various mental symptoms (called behavioral and psychological symptoms of dementia) appear. Depression is one of the symptoms and is thought often according to reports in Europe and the United States as frequent as 36.7% [1] to 47.8% [2]. On the other hand, drug treatments such as antidepressants should be cautiously used with risk of adverse events which may exceed their benefit [3]. Recent reports suggest that transcranial direct current electrical stimulation (tDCS) may be safe and effective as a treatment option.


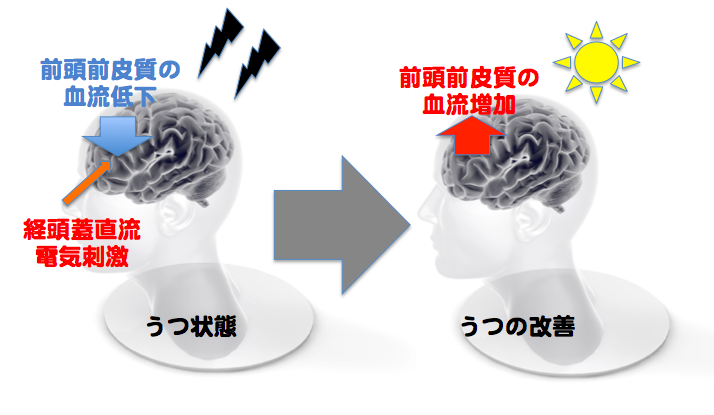


Figure 1. Hypothesis of tDCS working on the depression and blood flow

**What is transcranial direct current electrical stimulation (tDCS)?**

It is a device which stimulates the cranial nerve by applying a weak (2 mA in this study) current from the electrode attached to the scalp, various effects such as improvement of mental symptoms, improvement of cognitive function, increase of rehabilitation efficiency, etc. are expected. Regarding treatment of depression, the decrease in blood flow at the part of the frontal lobe called the frontal cortex on the left side (slightly above the forehead) is thought to be related to depressive symptoms, and the left prefrontal cortex is tDCS There is also a report that stimulus improves the depressed mood of depressed patients under 65 years old. [4, 5]

1. **Purpose of this study**

In this research, patients undergoing consent agree to undergo transcranial direct current electrical stimulation, measure and evaluate depressive symptoms, and examine their safety and effect. This research is to be carried out with the approval of the Ethics Committee of National Center of Neurology and Psychiatry.


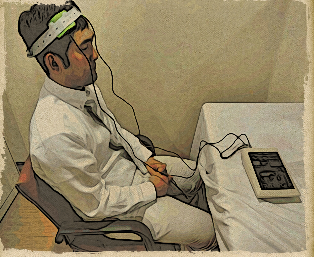


Figure 2. Principle investigator undergoing tDCS

1. **You have right to voluntarily participate and withdraw consent**

Please decide whether to participate in this research, depending on your free will. Even if you refuse to participate, for that reason you will not suffer any disadvantages for future treatment, your doctor will continue to do the best treatment with responsibility. Also, even after you have agreed to participate in this research, you can withdraw from participating in research freely without being disadvantaged at any time.

There is no disadvantageous response due to not agreeing to participate in this research and due to withdrawal

Even if you discontinue participating in this study, there is no disadvantage such as that the medical treatment for you will change.

1. **About research execution method and participation cooperation matter**

***Participants who can and cannot participate in the study**

(1) Patients who can participate

①If you are aged between 65 and 90

②If you have been diagnosed as Alzheimer’s disease

③If you have symptoms of depression according to the standardized test

④If you have been on the same antidepressants or have not taken any of them for at least two weeks.

⑤If you have been on the stable dose of donepezil, galantamine, rivastigmine or memantine or have not taken any of them for at least four weeks.

⑥If you are ambulant, or able to walk independently with or without any walking aids.

⑦Who have a study partner who can come to the clinic along with the participant and must spend at least 10 hours a week living with the participant.

⑧Who have a proxy (parents, adult sons and daughters, siblings or spouse) to get consent with.

(2) Participants who cannot participate

①If you simultaneously have dementia other than Alzheimer’s disease

②If you have evidence of mixed etiology (i.e., presence of other neurodegenerative or cerebrovascular disease, or another neurological or systemic disease or condition likely contributing to cognitive decline)

③If your physician decided to prescribe antipsychotics for their symptoms such as delusion, hallucination or agitation.

④If your physician considered your admission to hospital within six weeks due to depression or suicidal thought.

⑤If your physician considered that electroconvulsive therapy or tDCS had not been effective for your past depressive episodes.

⑥If you are clinically contraindicated to tDCS or electroconvulsive therapy.

⑦If you are currently on benzodiazepines and so-called Z drugs (zolpidem, zopiclone, and eszopiclone).

⑧If you are deemed as severe dementia according to standardized tests and clinical observation.

⑨If your severity of depression is too weak according to standardized tests.

⑩If you do not agree evaluation to the part of the video shoot of the interview to perform.

***Methods and time frame**

①First we interview individuals and study partners, and evaluate the function of living functions and the course of the disease.

②At the stage when participation is confirmed, participants are automatically assigned by computer to the group receiving tDCS, or the group receiving sham tDCS (wearing instruments, but practically no stimulation). It is not informed to the end to participants, proxy persons, study partners, evaluators who conduct psychological evaluation as to which way they are assigned.

③Following the schedule we will conduct the following inspections. Stimulation is completed after 15 stimuli. The participants will visit the hospital two weeks later and we will ask about the situation afterwards. It's about 5 to 6 weeks from beginning to end. Meanwhile, we will not only look at the effectiveness of tDCS but also check your medical condition and safety, so we ask you to keep the schedule properly.

④We will shoot video with part of the interview. Specifically, it is done at screening and at the end of 15 times. Time will be about 30 minutes for participants and study partners.

|  | Screening | After 5 stimuli | After 10 stimuli | After 15 stimuli | Follow-up |
| --- | --- | --- | --- | --- | --- |
| Consent | ● |  |  |  |  |
| Body hight and weight | ● |  |  |  |  |
| Interview with participants and study partners | ● |  |  |  |  |
| Physical examination | ● | ● | ● | ● | ● |
| Cognitive tests (participants/study partners) | ● |  |  |  |  |
| Cognitive tests(participants) | ● | ● | ● | ● | ● |
| Adverse events evaluation | ● | ● | ● | ● | ● |
| Depression assessments (participants) | ● | ● | ● | ● | ● |
| Depression assessments (study partners) | ● |  | ● | ● | ● |
| Neuropsychiatric symptoms evaluation (study partners) | ● |  |  | ● | ● |
| ADL evaluation (partners) | ● |  |  |  |  |
| Caregiver’s Burden evaluation (partners) | ● |  |  | ● | ● |
| Clinical global impression assessments (participants/study partners) |  | ● | ● | ● | ● |

***After study completion**

You are not able to be treated with tDCS since it is not clinically approved.

***Request for Participants, Representatives or Study Partners**

- Please contact us when you cannot visit the visit date.
- Please contact your research representative, investigators or clinical research coordinators beforehand if you change the method of taking medication taking or taking doses or change the contents of the ongoing therapy.
- While participating in this clinical study, please do not use drugs bought at pharmacies, the internet etc just by judgment of yourself or your family, please consult with your research representative, investigators or clinical research coordinators beforehand.

1. **Adverse events**

Adverse events (AEs) that may be caused by tDCS include the following symptoms from reports so far:

- AEs reported more frequently than 5%: skin redness, itchiness, tingling scalp, sleepiness, burning sensation of scalp, headache, neck pain, dizziness, acute mood change.
- AEs reported less frequently than 5%: fatigue, difficult to concentrate, nausea, diarrhea, delirium.

If such adverse events occur, the physician will see you and make appropriate interventions. From the results of previous studies, skin symptoms are often temporary, and it is said that it is often improved at the time of stimulation of the next day and there is no hindrance to research. The study discontinuation due to adverse events is reported as 1-2%.

Although no death cases have been reported, cases of delirium and mania are reported as a serious adverse event with a probability of 5% or less. If these serious adverse events occur, the physician will see you and make appropriate interventions.

1. **About privacy protection**

If you participate in this research, data on this research, such as specimens and medical information provided by you, will be managed by a number that is symbolized in a format that can not identify individuals. A correspondence table for matching the symbolized number and individual information is created. Information on this research, such as this correspondence chart and other personal information, was approved by the Ethics Committee of the Center in accordance with the "Medical Ethics Guidelines for Human Subjects (Partial Amendment to Heisei Heike 2009)" Under the management system, it is strictly managed and stored in the hospital.

1. **On the use of research results**

Since this research is conducted independently by our hospital, we will not provide research results to other research institutes.

With the result of this research, patent rights etc. will not occur.

1. **About cost participants and study partners pay**

With participation in this research, there is no cost burden on tDCS enforcement and psychological examination. For each psychological examination, 3000 yen is paid to each participant and partner as burden reduction cost, and 7000 yen is paid to participants for each stimulus of tDCS. The burden reduction cost will be transferred to their bank accounts.

1. **About disclosure of research plan and personal information**

If you wish, you can view the research plan of this research and the materials on the contents of personal information that can identify you acquired in the research, so please contact us.

1. **About dissemination of research results**

The results of this research will be announced only in academic fields such as academic presentations and papers. Even at the time of publication at that time, all personal information will be gathered after anonymizing, so your personal information will never be released.

1. **About funding source relating to this study**

This research is mainly funded by scientific research funds of the Ministry of Education, Culture, Sports, Science and Technology and Japan Society for the Promotion of Science (KAKEN: Grant-in-Aid for Young Scientists(B), No. 26860958). In addition, we have not received funds from companies and organizations with interests that could affect the reliability of the research. We declare no conflicts of interests to any profitable company.

1. **About clinical trial insurance**

Clinical research establishing new medical technology for medicines (and medical equipment) is indispensable for the development of medicine and medical care. However, it is difficult to completely prevent the occurrence of health damage caused by pharmaceuticals (or medical equipment) even if clinical research is properly carried out with full attention. In recent years, from the viewpoint of protecting patients and subjects who cooperated in research with good intentions for the development of medical medicine and medical care, emphasis is placed on countermeasures against health damage caused by pharmaceuticals and medical devices.

This clinical study will be scientifically planned and carefully done, but if you have any symptoms or physical disorders that are different from usual during your participation in this study, please notify your doctor immediately. We will immediately take appropriate measures and treatments. At that time, if you need exams and treatment, you will be charged with your health insurance as well as regular medical examination.

Also, in the unlikely event that a heavy health damage ("Death, 1st to 2nd Grade of Secondary Disorder (in compliance with the “Medical Side Effect Remedy System") arises due to this research, please refer to "Damage Insurance on Clinical Studies" which will pay the compensation fee. However, if the health damage occurs due to other reasons not related to this research, or if the cause / causal relationship of health damage can be clearly explained to others, or if your health damage is false, Or if you are seriously negligent, such as intentionally not obeying the instructions of a doctor, you may be unable to pay the compensation fee or the compensation fee may be restricted. The consent to participate in this clinical study does not mean that you waive the right to claim compensation in court.

1. **About agreement by surrogates**

Because this study covers patients with Alzheimer's disease, we included subjects that are difficult for us to fully understand and agree on ourselves. For that reason, we will explain to the surrogates such as your family to get consent, so we ask for your understanding and cooperation.

1. **Contact information**

About the study

Principle Investigator: Yuma Yokoi, MD
National Center of Neurology and Psychiatry

Department of Psychiatry

4-1-1, Ogawahigashi-cho, Kodaira-shi, Tokyo,

187-8551, Japan

Phone:+81-42-341-2711 (ext 3174)

Email: [yyokoi@ncnp.go.jp](mailto:yyokoi@ncnp.go.jp)

In case of emergency, please contact the research representative.

At nighttime and holidays, please call 042-341-2710 and tell them to connect with "Doctor in charge of psychiatry or doctor". At that time, please inform personnel that it is about "clinical study of tDCS".

Contact for complaints or ethical problems

National Center of Neurology and Psychiatry

Ethics Committee

4-1-1, Ogawahigashi-cho, Kodaira-shi, Tokyo,

187-8551, Japan

Email：[rinri-jimu@ncnp.go.jp](mailto:rinri-jimu@ncnp.go.jp)

Reference

1. Aalten, P., et al., Neuropsychiatric syndromes in dementia. Results from the European Alzheimer Disease Consortium: part I. Dement Geriatr Cogn Disord, 2007. 24(6): p. 457-63.

2. Acosta-Castillo, G.I., et al., [Neuropsychiatric symptoms in older adults with dementia and their relationship to disease severity]. Rev Invest Clin, 2012. 64(4): p. 354-63.

3. Banerjee, S., et al., Sertraline or mirtazapine for depression in dementia (HTA-SADD): a randomised, multicentre, double-blind, placebo-controlled trial. Lancet, 2011. 378(9789): p. 403-11.

4. Loo, C.K., et al., Transcranial direct current stimulation for depression: 3-week, randomised, sham-controlled trial. Br J Psychiatry, 2012. 200(1): p. 52-9.

5. Brunoni, A.R., et al., The sertraline vs. electrical current therapy for treating depression clinical study: results from a factorial, randomized, controlled trial. JAMA Psychiatry, 2013. 70(4): p. 383-91.
